# Distributed changes of the functional connectome in patients with glioblastoma

**DOI:** 10.1038/s41598-020-74726-1

**Published:** 2020-10-27

**Authors:** Karl-Heinz Nenning, Julia Furtner, Barbara Kiesel, Ernst Schwartz, Thomas Roetzer, Nikolaus Fortelny, Christoph Bock, Anna Grisold, Martha Marko, Fritz Leutmezer, Hesheng Liu, Polina Golland, Sophia Stoecklein, Johannes A. Hainfellner, Gregor Kasprian, Daniela Prayer, Christine Marosi, Georg Widhalm, Adelheid Woehrer, Georg Langs

**Affiliations:** 1 Computational Imaging Research Lab, Department of Biomedical Imaging and Image-Guided Therapy, Medical University of Vienna, Spitalgasse 23, 1090 Vienna, Austria; 2 Department of Biomedical Imaging and Image-Guided Therapy, Division for Neuro- and Musculo-Skeletal Radiology, Medical University of Vienna, Vienna, Austria; 3 Department of Neurosurgery, Medical University of Vienna, Vienna, Austria; 4 Division of Neuropathology and Neurochemistry, Department of Neurology, Medical University of Vienna, Vienna, Austria; 5 CeMM Research Center for Molecular Medicine of the Austrian Academy of Sciences, Vienna, Austria; 6 Department of Laboratory Medicine, Medical University of Vienna, Vienna, Austria; 7 Department of Neurology, Medical University of Vienna, Vienna, Austria; 8 A. Martinos Center for Biomedical Imaging, Massachusetts General Hospital, Harvard Medical School, Cambridge, USA; 9 Computer Science and Artificial Intelligence Lab, Massachusetts Institute of Technology, Cambridge, USA; 10 Department of Radiology, Ludwig-Maximilians-University, Munich, Germany; 11 Department of Medicine I, Medical University of Vienna, Vienna, Austria

**Keywords:** CNS cancer, Cancer in the nervous system

## Abstract

Glioblastoma might have widespread effects on the neural organization and cognitive function, and even focal lesions may be associated with distributed functional alterations. However, functional changes do not necessarily follow obvious anatomical patterns and the current understanding of this interrelation is limited. In this study, we used resting-state functional magnetic resonance imaging to evaluate changes in global functional connectivity patterns in 15 patients with glioblastoma. For six patients we followed longitudinal trajectories of their functional connectome and structural tumour evolution using bi-monthly follow-up scans throughout treatment and disease progression. In all patients, unilateral tumour lesions were associated with inter-hemispherically symmetric network alterations, and functional proximity of tumour location was stronger linked to distributed network deterioration than anatomical distance. In the longitudinal subcohort of six patients, we observed patterns of network alterations with initial transient deterioration followed by recovery at first follow-up, and local network deterioration to precede structural tumour recurrence by two months. In summary, the impact of focal glioblastoma lesions on the functional connectome is global and linked to functional proximity rather than anatomical distance to tumour regions. Our findings further suggest a relevance for functional network trajectories as a possible means supporting early detection of tumour recurrence.

## Introduction

Glioblastoma (GBM) is one of the deadliest types of cancer with profound impact on the individual patient’s neurocognitive function and quality of life^1^. After standard treatment, which consists of maximal safe resection followed by radio- and chemotherapy with the alkylating agent temozolomide, the vast majority of patients experiences early and abrupt tumour recurrence^2,3^. Recurring tumours are often extensive in size, more likely to involve both hemispheres, and quickly progress to terminal disease stages^4^. Thus, early detection and localization of tumour recurrence is key to initiate salvage treatment at an earlier stage.

Structural contrast-enhanced magnetic resonance imaging (MRI) is the gold standard for preoperative and longitudinal tumour mapping since it is non-invasive and offers high spatial resolution^5^. However, structural MRI does not appreciate the full extent of GBM as infiltrative margins are typically not visualized. Indeed, upon histology tumour cells of high proliferative potential and invasive capacities have been proven even far beyond the tumour edge within macroscopically normal brain parenchyma^6–8^. In addition to structural imaging, task-based functional MRI (fMRI) has been recognized as a relevant tool for preoperative mapping of eloquent cortical function^9^. However, task-based fMRI strongly depends on the patient’s compliance and ability to perform a task, which may be compromised by the brain tumour itself^10,11^. Hence, task-independent resting-state fMRI is gaining increased attention for its ability to evaluate functionally relevant areas even in impaired or non-compliant patients^12,13^. Indeed, resting-state fMRI-derived networks were found to exhibit stable characteristics within individual patients^14^, which renders them a promising candidate marker for longitudinal personalized evaluations.

Only recently, the global impact of brain tumours, even if circumscribed lesions, on brain networks has been acknowledged. For instance, higher alterations of the network structure were observed in high-grade as compared to low-grade gliomas^15^. Gliomas located in the left parietal cortex were found to be associated with widely reduced connectivity within the extended bilateral default mode network^16^, unilateral gliomas were found to disrupt long-range inter-hemispheric connectivity^17^, and restricted lesions to induce a global reconfiguration of network complexity^18^. Conversely, spatially distributed lesions might impact the same functional networks and produce identical neurological symptoms^19,20^. Hence, the precise spatio-temporal interplay of tumour, network alteration, and functional compensation/reorganization is just beginning to be understood^21,22^.

**Figure 1:**
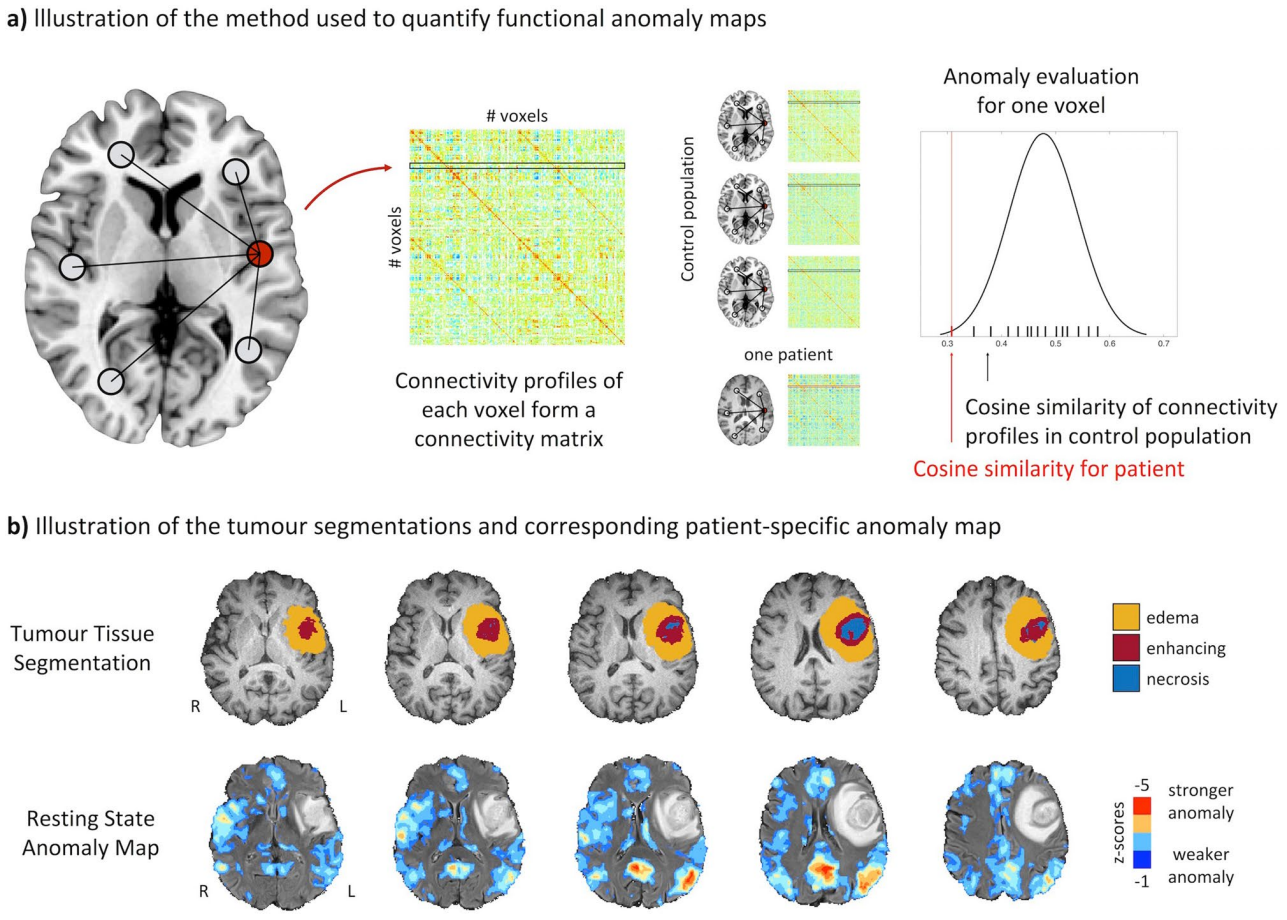
Illustration of the method used to quantify patient- and voxel-wise anomaly maps. (**a**) Based on a control group of 80 individuals, for each voxel a baseline cosine similarity between the voxel-wise connectivity patterns is calculated. Patient specific anomaly maps are generated by quantifying the connectivity profile deviation from the control group. (**b**) Illustration of the generated tumour tissue segmentations and a patient-specific anomaly map. Figure created with ITK-SNAP 3.8 (www.itksnap.org), MATLAB 2014a (www.mathworks.com) and Microsoft Office PowerPoint 2016 (www.microsoft.com).

In this work, we hypothesized that network anomalies are not restricted to areas of spatial tumour involvement but distributed more globally and follow a functional rather than a spatial distance to the tumour. To answer this question, we quantified tumour-induced network anomalies of patients with GBM based on their deviation from network connectivity of a healthy control population (Fig. 1) and disentangled their spatial and functional relations to the brain tumour.

## Results

### Functional network anomaly follows functional rather than spatial distance to the tumour

**Figure 2:**
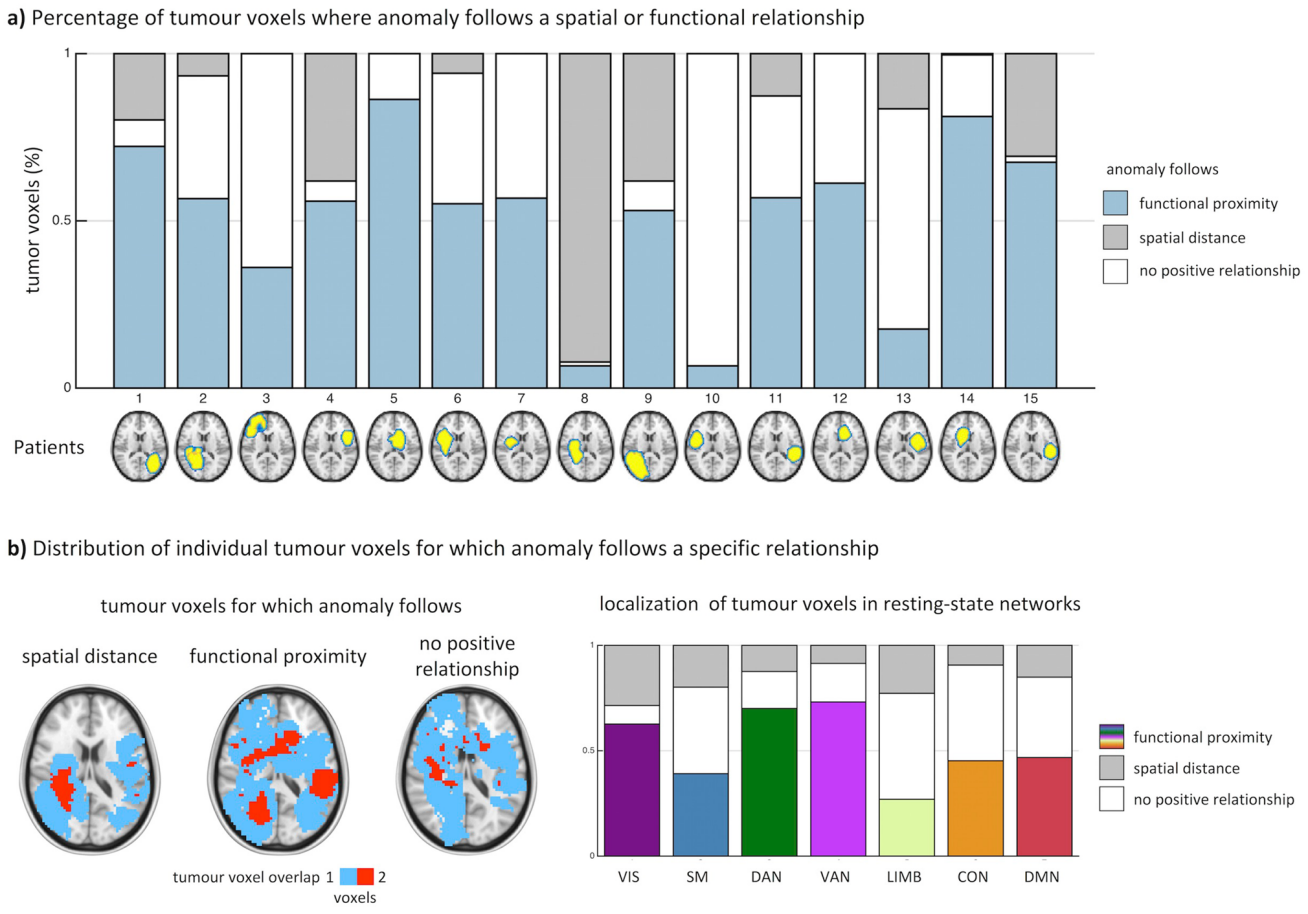
Anomalies are situated along functional proximity to tumour voxels rather than spatial distance. (**a**) In 14 out of 15 patients, a higher percentage of tumour voxels is observed where the anomaly follows functional proximity rather than spatial distance. (**b**) Tumour voxels, for which anomalies relate to spatial distance are primarily found in spatially compact networks such as the visual (VIS), somatomotor (SM), or limbic (LIMB) networks. Tumour voxels for which a functional proximity relates to anomaly are primarily found in higher order networks such as the dorsal (DAN) and ventral attention networks (VAN), default mode (DMN) as well as the frontoparietal control (CON) network. Figure created with MATLAB 2014a (www.mathworks.com) and Microsoft Office PowerPoint 2016 (www.microsoft.com).

We assessed functional network anomaly using anomaly scores as quantitative measures. In 14 out of 15 patients, we observed highly anomalous voxels with anomaly scores less than − 2.3 preferentially distributed along their functional distance to tumour-bearing voxels rather than their spatial distance (Fig. 2a). A higher number of voxels (paired t-test: t(14) = 2.92, p = 0.0111) as well as a higher percentage of the entire tumour volume (paired t-test: t(14) = 3.18, p = 0.0066) was observed where anomalies relate to functional proximity (Supplementary Fig. S1). Specifically, across all patients and for all seven resting-state networks, we found that functional proximity to the tumour correlated stronger with anomaly than spatial distance (Fig. 2b). Tumour voxels associated with closer functional proximity to anomalies were mainly found in brain regions belonging to higher-order larger functional networks such as the dorsal or ventral attention networks, but also to some extent the visual network. Compared to higher-order networks, spatial distance was more closely correlated to network anomaly in tumours that were at least in parts located within smaller spatially restricted networks such as the visual, somatomotor and limbic networks. The visual network exhibited both, tumour voxels associated with functional proximity and spatial distance.

### Functional network anomaly is symmetric and mirrored in the cerebellum

**Figure 3:**
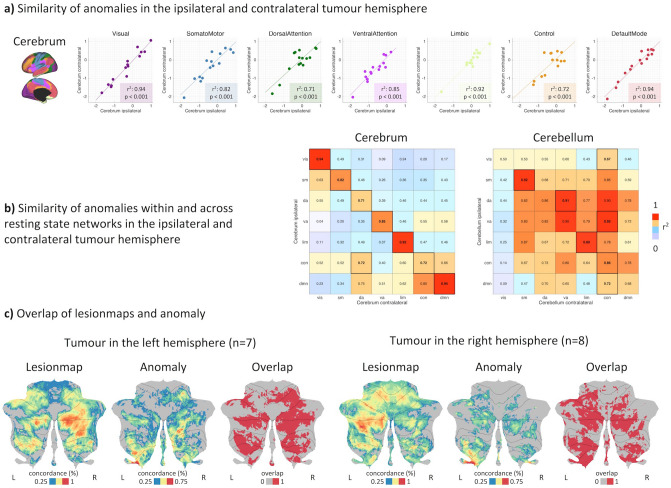
Distribution of anomaly scores in seven resting-state networks. (**a**) The patient-specific anomaly scores showed a symmetry within cerebral resting-state networks in the ipsilateral and contralateral tumour hemispheres. (**b**) Within and across resting-state network correlation of anomaly scores revealed symmetric alterations particularly in the cerebrum. For the cerebellum, similarity was not restricted to the same resting-state networks on both hemispheres, indicating a disturbed cerebellar connectivity structure. (**c**) Patients with a tumour located in the left hemisphere showed a higher overlap between lesion-map and anomalies (thresholded at p = 0.05 uncorr.) in the contralateral (right) cerebellum. The same effect was observed in patients with a tumour located in the right hemisphere, where the contralateral (left) cerebellum showed a higher overlap between lesion-map and anomaly (thresholded at p = 0.05 uncorr.). Figure created with MATLAB 2014a (www.mathworks.com) and Microsoft Office PowerPoint 2016 (www.microsoft.com).

Interestingly, network anomaly was found to be highly symmetric across both cerebral and cerebellar hemispheres irrespective of being ipsi- or contralateral to the tumour. This association was true for all seven resting-state networks (Fig. 3a). Looking at individual networks, the default mode and visual networks exhibited the highest symmetry (r^2^ = 0.94, p < 0.001), whereas a lower but still significant correlation (r^2^ = 0.71, p < 0.001) was found for the dorsal attention network (Fig. 3a). Bi-hemispheric correlations were higher within the same than across different networks except for the frontoparietal control network that showed an equally high correlation to the contralateral dorsal attention network (r^2^ = 0.72) (Fig. 3b).

As supratentorial resting state networks are also represented in the cerebellum in a crossed hemispheric way, we independently evaluated the presence of propagated network anomaly within cerebellar resting state networks and again found symmetric anomaly, even though the bi-hemispheric correlations were not as network-specific as within the cerebrum (Fig. 3b). For both left and right hemispheric tumours, results showed a higher overlap between lesion-maps (i.e., regions that are functionally connected to tumour locations in controls) and network anomalies in the contralateral cerebellar hemisphere (Fig. 3c). This association was observed across a range of network anomaly thresholds (Supplementary Fig. S2). In other words, patients with a tumour located in the left cerebral hemisphere showed a higher correlation between lesion-map and network anomaly in the contralateral (right) cerebellar hemisphere (paired t-test: t = 15.92, p < 0.001). The same association was observed in patients with a tumour located in the right hemisphere (paired t-test: t = 11.77, p < 0.001).

### Decrease of network anomaly at first follow-up after glioblastoma surgery

**Figure 4:**
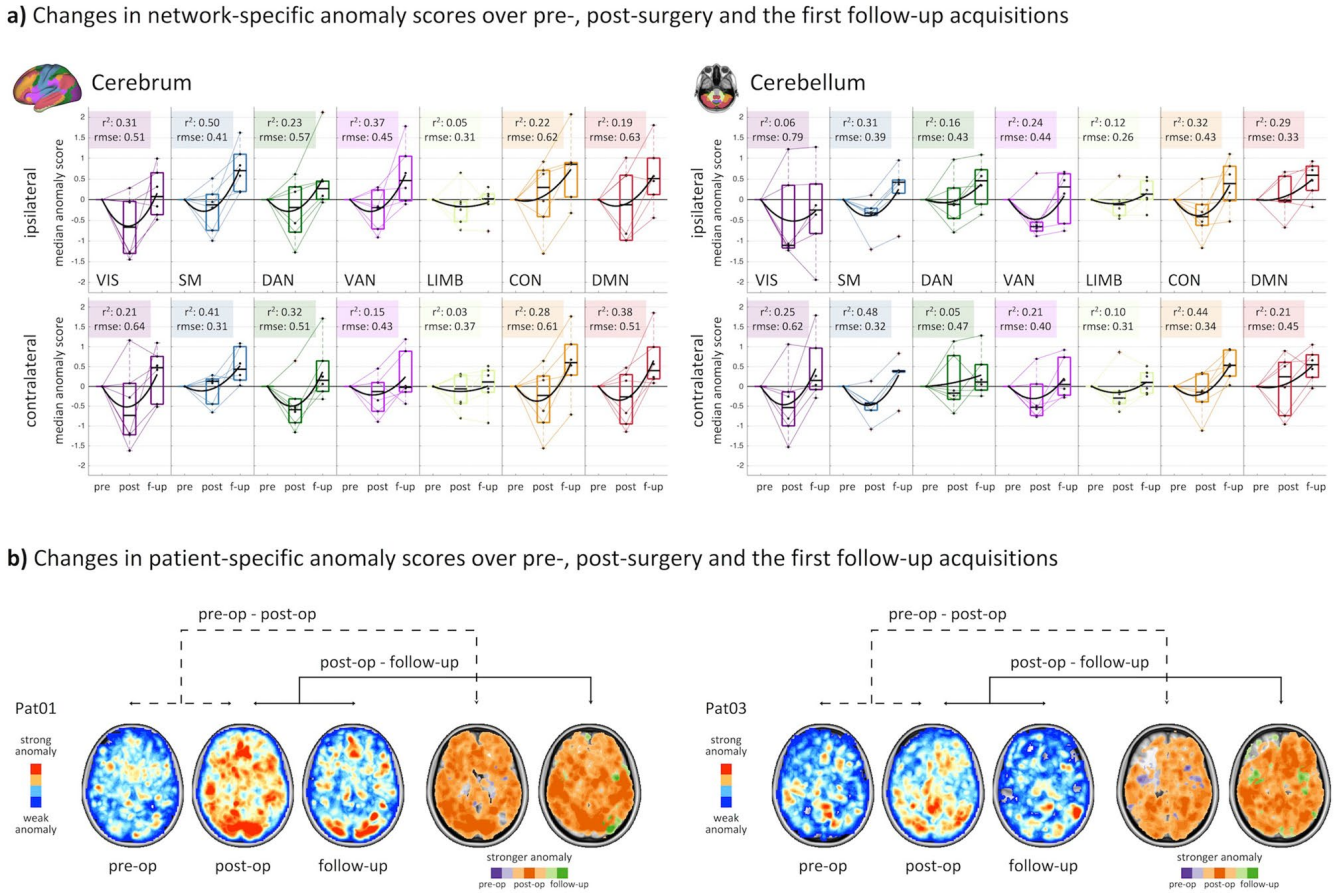
Longitudinal anomaly trajectories for pre-, post-surgery and the first follow-up. (**a**) Evaluation of the anomaly score trajectories over pre-, post-surgery and the first follow-up scans showed a decrease of network anomaly at first follow-up after glioblastoma surgery for all resting-state networks. (**b**) Maximum intensity projections of patient-specific anomaly score trajectories over pre- and post-surgery to first follow-up acquisitions. Figure created with MATLAB 2014a (www.mathworks.com) and Microsoft Office PowerPoint 2016 (www.microsoft.com).

Longitudinal trajectories of the individual patient’s network anomalies revealed a recovery in form of a decrease in network anomaly at the first follow-up two months after surgery (Fig. 4, Supplementary Fig. S3). This characteristic chronology was observed in the cerebrum and cerebellum. In contrast to the highly symmetric network alterations at time of diagnosis, regression analysis revealed distinct lateralization of r-squared values characterizing the trajectories. Some networks showed preferential ipsilateral involvement (visual and somatomotor networks) while others showed more pronounced trajectory in the contralateral hemisphere (dorsal attention, default mode network). For both scenarios, the lateralization of the higher r-squared values of longitudinal network alterations was mirrored in the cerebellum.

### Network anomaly precedes tumour recurrence

**Figure 5:**
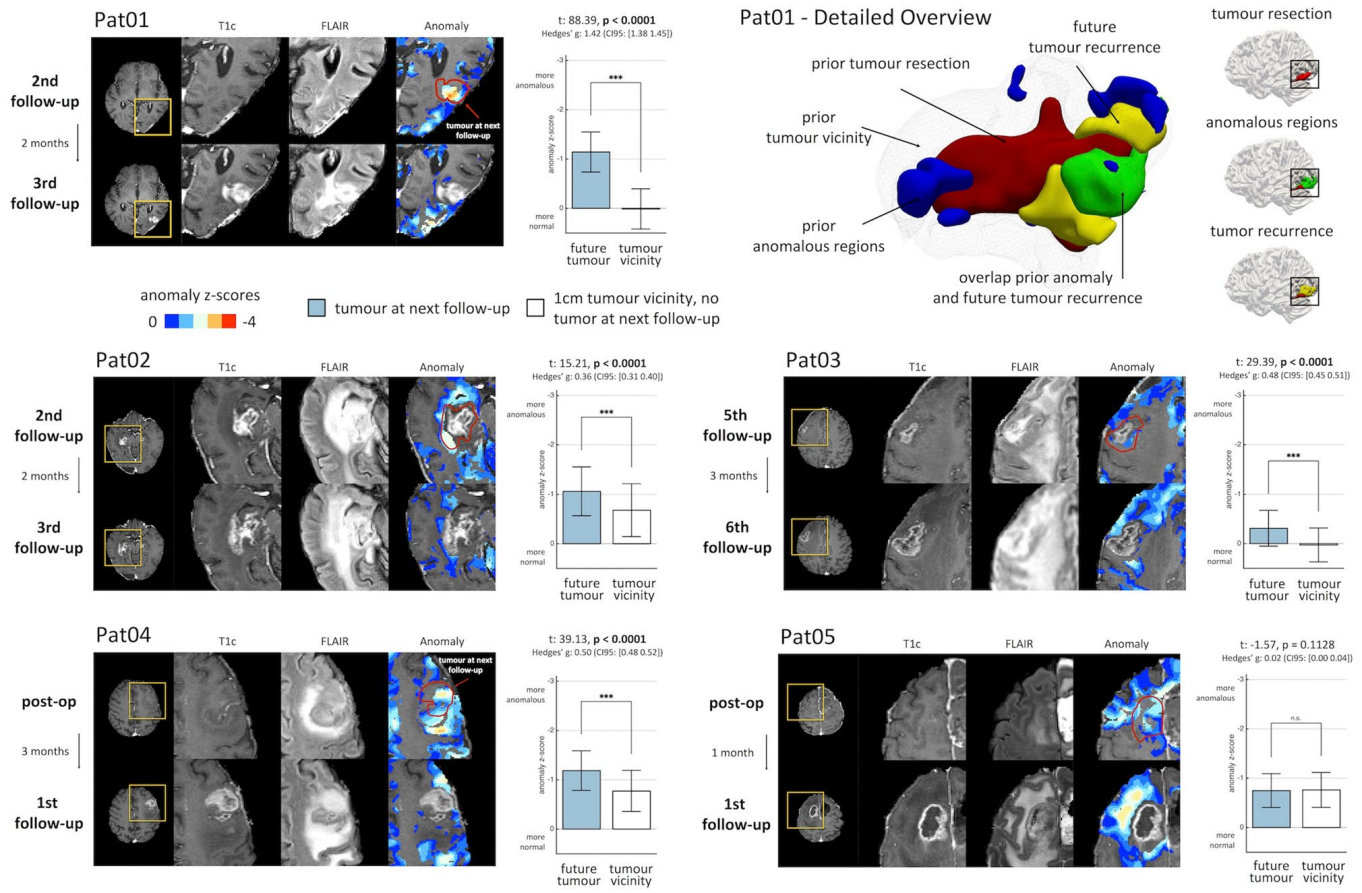
Tumour recurrence coincided with functional anomaly before structural changes became apparent on structural imaging. (**a**) In Patient 01, regions of tumour recurrence at the third follow-up exhibited anomalous connectivity patterns already 2 months earlier at the second follow-up examination. Compared to all other voxels in the tumour vicinity, future tumour voxels were significantly more anomalous (p < 0.0001). (**b**) A detailed 3d illustration of the same patient showing the resection zone, tumour recurrence and overlap with the regions with aberrant connectivity profiles (anomaly z-score < − 1 for illustration). (**c**–**e**) In Patient 02, Patient 03 and Patient 04, regions of future tumour recurrence and progression showed a higher network anomaly compared to the structurally affected tumour vicinity (p < 0.0001). (**f**) In Patient 05, emerging tumour regions were mainly observed in the white matter and were not associated with significant preceding network anomaly (p = 0.1128). Figure created with ITK-SNAP 3.8 (www.itksnap.org), ParaView 5.5 (www.paraview.org), MATLAB 2014a (www.mathworks.com) and Microsoft Office PowerPoint 2016 (www.microsoft.com).

Finally, we compared the timing of (re-)occurrence of network anomaly and recurrence of contrast-enhancing tumour in five patients where we had extended longitudinal data (Fig. 5). Importantly, network anomaly preceded enhancing tumour recurrence by two months in four out of five patients, whose tumour recurrence involved the grey matter. Moreover, network anomaly was significantly higher at the exact location of subsequently emerging tumour as compared to a 1 cm rim surrounding the entire resection cavity. In contrast, one patient whose tumour recurrence was primarily in the white matter (Patient 05) did not show the characteristic preceding network anomaly. In the four patients with recurrence in the grey matter, voxel-wise prediction of future contrast-enhancing tumour yielded a significantly higher area under the curve (AUC) than chance (p < 0.0001 uncorrected), where in Patient 01 we observed good discrimination (AUC = 0.85) and in 3 patients we found moderate discrimination (AUC ≥ 0.60). In Patient 05, with future tumour voxels primarily in the white matter, we observed a prediction performance similar to chance (AUC = 0.50, p = 0.3496). Figure 5 illustrates the timing and location of network anomaly and subsequently emerging tumour recurrence for all 5 patients. A detailed description of the individual patients’ anomaly and recurrence trajectories is provided in the Supplemental material and in Supplementary Fig. S5.

## Discussion

We used resting-state fMRI to evaluate changes in the functional network structure of 15 patients with GBM at time of diagnosis and longitudinally tracked changes during treatment and follow-up in a subset of patients. We found patterns of network alterations across all patients irrespective of tumour size or location. In all cases, focal lesions were associated with widespread and symmetric anomaly in both hemispheres in a network-specific manner across all seven resting-state networks in line with recent work^15,23^. In our study, network anomaly refers to the *relative deviation* from network connectivity of a control population and does not necessarily reflect increased or decreased activity. To ensure that the observed global alteration of networks was not simply due to tumour-associated destruction and loss of axonal connections, we excluded all tumour-bearing voxels when calculating network anomaly scores. Thus, the reported changes are observed in connections not directly destroyed by the tumour. Vice versa, inclusion of all directly tumour voxel-related connections did not change the observed patterns of network anomaly, which supports our hypothesis of a global tumour impact on the entire network architecture. Even though the exact mechanisms remain speculative, such a global impact might be due to forward-propagation of locally perturbed connections within one network (such as tumour-associated destruction and oedema) to hierarchically connected further networks. Of note, the global impact we observe is in line with recent work that also included tumour-bearing voxels in their analysis^15^. Our results corroborate recent studies which observed a shift in connectivity profiles in patients with newly diagnosed glioma^23^. Especially high-grade gliomas showed increased disturbances, accompanied by altered hub-non-hub connectivity. It is also in line with the concept of GBM being a disease that involves the entire brain, which is best exemplified by its highly invasive growth capacity with single tumour cells that invade the most distant brain regions^8,24^. Indeed, a more extensive impact on neurologic function and network connectivity than merely explained by the macroscopic mass lesion has been documented for interhemispheric connections^18,25,26^. Here, we advance this concept of GBM as a whole brain disease by highlighting the fact that tumour-related network alterations may be spatially widely distributed as long as they are functionally close. In fact, we found functional proximity to even dominate the pattern of involved resting-state networks, while spatial distance in the anatomical space did matter to a lesser extent. This is especially true for within-network propagations to the contralateral hemisphere, which leads to a systematic and regular involvement of both cerebral hemispheres.

Symmetric involvement is in line with a brain geometry that is defined by functional connections as a relevant alternative to an anatomical space^27^. The relatively larger impact of functional tumour proximity on network anomaly was especially evident in the spatially distributed higher-order networks such as the dorsal and ventral attention networks. In contrast, spatial distance seemed more relevant in tumours that involved spatially restricted networks such as the visual, limbic, or somatomotor networks. Reassuringly, network anomalies were mirrored in the contralateral cerebellum, as previously shown for task-based brain activation^28,29^ and surgery-related alterations^30^. In concordance with these studies, our findings suggest that cerebellar network activity is readily altered by focal cerebral lesions, albeit not necessarily mediated through structural connectivity (as direct tumour connections were excluded in anomaly scores).

From a clinical perspective, several studies have observed a considerable neurocognitive deterioration in patients with glioma^25,31–34^, and cognitive decline has been found to precede MR imaging-defined tumour recurrence^35^. The widespread network anomalies that we observed might hint at the neural basis of those deficits, however no neurocognitive assessment was possible in our cohort and thus the relationship remains speculative. A more detailed assessment of the individual patient’s neurocognitive function as part of the routine patient care is warranted and might also add to our current understanding of disease-related quality of life. Moreover, the striking symmetry of network anomaly was independent of right- or left-hemispheric tumours and contrasts clinically relevant lateralization of neurologic functions such as language, which suggests an important, so far neglected role also for the non-dominant hemisphere.

So far, only few studies have investigated the impact of neurosurgery on the brain’s functional network structure. Together they suggest a transient decrease in spontaneous neuronal activity in the cerebellum and reduced connectivity among default mode regions or interhemispheric connections of motor areas, all of which typically resolve within 3 months^30,36,37^. Our findings corroborate previous results and first demonstrate near total or supra-total recovery of functional network activity upon GBM surgery. The short time period until recovery at first follow-up (i.e., two months) suggests transient and reversible alterations mediated by the tumour and surgical intervention rather than true plasticity of network architecture.

A major strength of our approach is the ability to follow individual patient’s network trajectories in parallel to structural tumour evolution beyond perioperative phases throughout the entire disease course. By doing so we observed consistent and specific network alterations at locations, where subsequent tumour recurrence became evident two months later. Even though not all regions with altered connectivity, especially remote from the original tumour site, necessarily corresponded to areas of future tumour growth, the consistency and predictive value of preceding network anomaly in four out of five patients suggests that resting-state networks are sensitive to microscopic tumour growth and/or increased oxidative demand, thereby rendering resting-state fMRI a promising marker for the early detection of tumour recurrence. Similarly, other imaging methods such as PET or magnetic resonance spectroscopy add value to the visualization of invasive margins beyond structural imaging^38,39^. However, in contrast to those techniques, resting-state fMRI enables fast, cost-effective, and non-invasive prediction of tumour growth without the need for radioactive tracers and specialized imaging units. Given the exceedingly poor post-recurrence survival of patients with GBM, the ability to predict tumour growth by two months will impact the clinical scenario by bringing second surgeries and/or adjuvant treatments forward to a point in time, when the disease is still localized. This will ultimately result in a higher rate of second remissions and more individualized patient management. However, future research is necessary to determine the causes that underlie network anomaly and dissect the relative contributions of tumour infiltration, oedema/reactive changes, neurovascular uncoupling^40^ or other biochemical effects^38^.

Our study has limitations. First and most important, the sample size of our cohort is limited and continued assessments in larger patient cohorts with additional focus on longitudinal measurements will be crucial to precisely define the role of resting-state fMRI in advancing our understanding of varied neurologic/neurocognitive phenotypes of brain tumour patients, as well as translating it to routine clinical practice. In this regard, a comparison also to other brain tumour types will be helpful to better delineate characteristics unique to patients with GBM and differentiate them from other infiltrative and non-infiltrative brain tumours such as meningiomas or metastases. For the moment, we decided to focus on patients with primary glioblastoma (as histologically and molecularly defined) to ensure a homogeneous tumour behavioural background given variable tumour locations. Also, larger multicentre studies that incorporate different MR scanners and sequences will be needed to establish the analytical performance of resting-state network anomaly as a potential biomarker. Along this line, image analysis of brain tumour patients is especially challenging due to the structural lesions the tumour may cause. In particular, it might be impossible to reliably align and quantify diseased brains, which prompted us to manually inspect and adapt all pre-processing steps carefully to make sure not to introduce any errors. The network anomaly maps we created are based on functional connectivity, which per se is primarily a function of higher cortical areas. Hence, they may not reliably detect aberrations due to subcortical white matter lesions (as highlighted by the inability to detect the tumour recurrence in our fifth longitudinal patient). Still, our single case-based observation is too premature to confirm or rule out a relevant association. Finally, and most importantly, future studies will benefit from addition of more detailed and time-consuming neuropsychological assessments since the observed global network anomalies suggest global neurocognitive alterations. Unfortunately, advanced neuropsychological data were not available for our initial cohort. Similarly, the restricted sample size with variable tumour locations, lesion sizes, and associated neurological symptoms prevented us from further subgroup analyses based on functional or spatial lesion overlap.

In summary, we assessed network anomaly in patients with GBM and found widespread, highly symmetric network alterations that follow a functional rather than spatial proximity to the tumour and are mirrored in the cerebellum. We further show that network deterioration is ameliorated within two months after neurosurgery. Ultimately, we find network alterations to precede structural tumour recurrence by two months, which highlights the potential of resting-state network anomaly as clinically relevant marker for the early detection of GBM recurrence.

## Methods

### Study cohort

The study cohort consists of 15 consecutive patients (61.2 + /- 10.1 years) with newly diagnosed GBM, IDH wildtype, WHO grade IV to ensure a more homogeneous tumour background. Six patients were longitudinally followed using bi-monthly structural MRI and resting-state fMRI. Tumour progression was defined according to response assessment in neuro-oncology (RANO) criteria^5^, by determining the increase in contrast-enhancing and non-enhancing FLAIR/T2W lesions, or the occurrence of any new lesions. An overview of the study cohort, primary tumour location, tumour extent and the number of longitudinal follow-up scans is given in Supplementary Table 1. The study was approved by the Ethics Committee of the Medical University of Vienna (reference number 1315/2015), and all participants provided written informed consent according to the Declaration of Helsinki after the design of the study were explained.

As a control cohort we used the University of McGill subset of a larger publicly available dataset^41^. This dataset comprises resting-state fMRI scans of 80 healthy individuals (aged 65.3 + /- 6.2 years), acquired at the same MR scanner type (Siemens TrioTim 3.0 T MRI, equipped with a 12-channel head coil), comparable EPI sequence (TR/TE 2000/30 ms, flip angle 90°, field of view 256, matrix size 64 × 64, voxel size 4 × 4 × 4 mm, 32 slices), and resting-state condition (individuals were instructed to relax, not to fall asleep and to keep their eyes closed).

### MRI acquisition

MRI data were acquired using a Siemens TrioTim 3.0 T MRI, equipped with a 12-channel head coil, at the Department of Biomedical Imaging and Image-guided Therapy, Medical University of Vienna. The MR imaging session comprised acquisition of high resolution structural images, including T1-weighted sequences (TR/TE 1650/3.71 ms, flip angle 8°, voxel size 1 × 1 × 1 mm) pre- and post-intravenous contrast media application (0.1 mmol/kg body weight of a gadolinium-based contrast agent), a T2-weighted sequence (TR/TE 5900/109 ms, flip angle 149°, voxel size 0.9 × 0.9 × 3 mm), and a FLAIR volume (TR/TE 9220/100 ms, flip angle 150°, voxel size 0.9 × 0.9 × 4 mm). For functional MR images an EPI sequence was employed (TR/TE 1980/30 ms, flip angle 90°, field of view 192, matrix size 64 × 64, voxel size 3 × 3 × 3.75 mm, 32 slices) with a total scan time of 10 min. Patients were instructed to relax, not to fall asleep and to keep their eyes closed.

### Data processing

Data pre-processing was performed with FSL v5^42^. First, the structural T1 volume was skull-stripped and segmented into grey matter (GM), white matter (WM) and cerebrospinal fluid (CSF). Then, the T1 volume was nonlinearly registered to the Montreal Neurological Institute (MNI) template using deformable registration via attribute matching and mutual-saliency weighting (DRAMMS)^43^. DRAMMS incorporates mutual saliency weighting to account for missing correspondences or the structural impact of pathologies such as brain tumours. All normalizations were carefully checked for errors, and the acquired normalization was applied to the segmented brain tissues. The fMRI data was motion corrected, where the functional volumes were registered to the mean volume, and framewise displacement (FD) was calculated to quantify motion outliers. The fMRI data was co-registered to the anatomical T1 volume, and the transformation obtained by DRAMMS was applied to normalize the fMRI data. Denoising was performed with ICA-Aroma^44^ with subsequent nuisance regression including the average WM, CSF and global signal as well as their first derivatives as regressors. Additionally, high-pass filtering with 0.01 Hz was applied, and no low-pass filtering was conducted^45^. The fMRI volumes were resampled to 3 × 3x3 mm voxel size, and spatially smoothed with 4 mm full width half maximum (FWHM). High motion time points with a framewise displacement > 0.5 mm were censored^46^. A liberal grey matter mask was created based on the control cohort resulting in approximately 60,000 voxels. Voxel-wise functional connectivity was quantified with Fisher z-scored Pearson’s correlation coefficient.

### Tumour segmentation

Automated tumour segmentation was performed with Bratumia^47^. The four structural image acquisitions (i.e., pre- and post-contrast T1-weighted, FLAIR and T2-weighted images) were used to quantify four distinct tumour tissue classes: necrotic tumour, enhancing tumour, non-enhancing tumour, and oedema. Tumour segmentations were manually inspected and corrected for segmentation errors. Additionally, resected tumour regions were manually delineated and added to the segmentation. Tumour segmentations were normalized with the transformation obtained by DRAMMS and smoothened with 3 mm FWHM to ensure a liberal tumour masking.

### Quantifying similarities in connectivity profiles

For each patient we quantified the voxel-wise similarity in connectivity patterns and its deviation from the control population (Fig. 1a). First, for each of the 80 controls a voxel-wise functional connectivity matrix was generated by calculating Pearson’s correlation between the time-series of each voxel. Then, a group average reference connectivity matrix was established by taking the mean of all 80 individual connectivity matrices. Subsequently, we quantified the voxel-wise similarity for all individuals in the control cohort in relation to the reference connectivity matrix. For this, we follow previous work that utilizes similarity of positive connectivity patterns^48,49^ and calculated the row-wise cosine similarity between each individual connectivity matrix and the reference connectivity matrix using only positive correlations. We discarded negative correlation values and used only positive relations to obtain interpretable values ranging from zero (dissimilar connectivity pattern) to one (highly similar connectivity pattern). This resulted in patient-specific vectors, where each entry quantifies the voxel-wise similarity of the connectivity profiles, providing a distribution of similarity measures across the control cohort for each voxel. We then characterized this distribution by quantifying the median and the median absolute deviation (MAD), which allowed us to calculate a patient-specific voxel-wise robust measure of dispersion^50^.

### Patient-specific anomaly maps

For each patient and resting-state acquisition we calculated the similarity to the reference control connectivity matrix and projected the resulting similarities into the control baseline distribution by subtracting the median and dividing it by the MAD (Fig. 1b). Subsequently, we projected the resulting maps back to the patient-space. This provided a patient-specific, voxel-wise score of functional connectivity deterioration, which we refer to as *anomaly score*.

For each patient, individual tumour voxels were excluded from the patient-specific connectivity matrices as well as for the control connectivity profiles. We calculated an individual baseline distribution for each patient, removing the affected patient-specific tumour voxels also in the control cohort. We removed voxels labelled with any of the four segmented tumour tissue types or segmented as resection. Tumour segmentations were slightly smoothed to make sure not to include any voxel directly affected by the pathology. Although anomaly maps calculated with and without tumour voxels were highly correlated (Supplementary Fig. S4), we removed all tumour voxels to avoid any potential influence of structurally abnormal voxels.

### Analyses

To evaluate the impact of a brain tumour on distinct resting-state networks we used a cortical delineation of seven well described supratentorial resting-state networks^48^ and their representation in the cerebellum^51^. Those resting-state networks included visual, somatomotor, dorsal attention, ventral attention, limbic, fronto-parietal control and default mode networks.

#### The relationship between anomaly and functional proximity or spatial distance

We analysed whether aberrant connectivity patterns followed a spatial distance or a functional proximity to the tumour. For each patient and tumour voxel (labelled as enhancing or necrotic tumour tissue) we quantified the Euclidean distance (*spatial distance*) and the absolute correlation of the average connectome in controls (*functional proximity*) to all other non-tumour voxels. Precisely, for each tumour voxel we calculated Pearson’s correlation between the distances and the anomaly scores of non-tumour voxels, and quantified whether a shorter anatomical distance or a stronger functional proximity relates more to higher anomaly. We compared positive relationships between distance and anomaly for functional proximity and spatial distance respectively. Voxels were excluded from comparison in case no positive correlation was observed and labelled as “no positive relationship”. Of note, to exclude effects driven by impaired connections to tumour-bearing regions, connectivity values between tumour and non-tumour voxels did not enter the calculation of the anomaly maps. For each patient, we calculated the percentage of tumour voxels to which highly anomalous voxels (z-score < − 2.3) rather follow a spatial or a functional distance.

#### Similarity of resting-state network anomaly in cerebrum and cerebellum

For each patient and resting-state network, we quantified the median anomaly score for ipsilateral and contralateral tumour hemispheres in the cerebrum, and for the corresponding networks in the cerebellum. We calculated the correlation coefficient of the anomaly scores between the hemispheres within each network and across different networks.

#### Tumour representation in the cerebellum

We quantified whether the tumour was represented in the cerebellum and whether this representation coincided with tumour-induced functional anomalies. For each patient and tumour voxel, we calculated lesion-maps^19,20^, i.e., all regions connected to patient-specific tumour-bearing voxels in controls. We then calculated the overlap of those lesion-maps and correlated them with the overlap of anomaly scores over a range of thresholds to determine threshold-independent concordance. We evaluated left and right hemispheric tumours separately and the overlap for ipsi- and contralateral hemispheres in the cerebellum and cerebrum.

#### Surgical impact on the connectivity structure

For the longitudinal patient cohort, we evaluated individual trajectories of anomaly scores for the first three resting-state acquisitions. For pre-, post-surgical and first follow up scans, we quantified the median network anomaly score for resting-state networks in the cerebrum and cerebellum, ipsi- and contralateral to the tumour. The pre-surgical time-point was used as baseline, and for each patient we subtracted the baseline anomaly score to align patient-specific trajectories. We performed second order polynomial regression and calculated the r-squared value and the root mean squared error (RMSE) to characterize the fit.

#### Relation between functional anomaly and tumour recurrence

To explore the relationship between network anomaly score and structural tumour evolution, we evaluated the coincidence of future tumour regions and anomaly scores at the acquisition time-point before enhancing tumour progression or recurrence was apparent upon structural imaging. One patient (Patient 06) had to be excluded from further analysis since it was not feasible to precisely quantify enhancing tumour tissue at time of follow-up scan due to motion artefacts during the contrast-enhanced T1-weighted image acquisition.

We compared the anomaly scores of future tumour regions to anomaly scores of voxels which were spatially within a 1 cm margin to the previous tumour or resection cavity, which we refer to as “tumour vicinity”. Tumour vicinity provides a more appropriate baseline for comparison than the overall average brain due to potential tumour infiltration^52^ or post-surgical effects. Between two consecutive follow-up scans t0 and t1, we quantified the enhancing and necrotic tumour present at time-point t1 but not at time-point t0. Subsequently, we compared the network anomaly scores between these prospectively emerging tumour voxels and all other voxels in the tumour vicinity with a two-sample permutation test. To exclude any bias introduced by segmentation errors, we analysed only follow-up scans where at least 3 cm^3^ were labelled as new enhancing tumour at time-point t1.

### Statistical analysis

All statistical tests were based on permutation-based testing, appropriately corrected for multiple comparisons using the false discovery rate (FDR) procedure^53^ to restrict the proportion of false positive findings to 5% (q = 0.05).

## Supplementary information

Supplementary information.

## Data Availability

The data and functional anomaly maps generated and analysed during the current study are available from the corresponding author upon request.
