# Supplementary material for: Distributed changes of the functional connectome in patients with glioblastoma
Source: Sci Rep. 2020 Oct 27;10:18312. doi: 10.1038/s41598-020-74726-1 (PMC7591862; doi:10.1038/s41598-020-74726-1)
Supplement: Supplementary file 1 — Supplementary information. [file 41598_2020_74726_MOESM1_ESM.pdf]

Supplementary Information

Distributed changes of the functional connectome in patients with glioblastoma

Karl-Heinz Nenning[1]*†, Julia Furtner[2]†, Barbara Kiesel[3], Ernst Schwartz[1], Thomas Roetzer[4], Nikolaus Fortelny[5], Christoph Bock[5], Anna Grisold[6], Martha Marko[6], Fritz Leutmezer[6], Hesheng Liu[7], Polina Golland[8], Sophia Stoecklein[9], Johannes Hainfellner[4], Gregor Kasprian[2], Daniela Prayer[2], Christine Marosi[10], Georg Widhalm[3], Adelheid Woehrer[4]◆, Georg Langs[1,8]*◆

† and ◆ contributed equally

1 Department of Biomedical Imaging and Image-guided Therapy, Computational Imaging Research Lab, Medical University of Vienna, Vienna, Austria
2 Department of Biomedical Imaging and Image-guided Therapy, Division for Neuro- and Musculo-Skeletal Radiology, Medical University of Vienna, Vienna, Austria
3 Department of Neurosurgery, Medical University of Vienna, Vienna, Austria
4 Division of Neuropathology and Neurochemistry, Department of Neurology, Medical University of Vienna, Vienna, Austria
5 CeMM Research Center for Molecular Medicine of the Austrian Academy of Sciences, Vienna, Austria
6 Department of Neurology, Medical University of Vienna, Vienna, Austria
7 A. Martinos Center for Biomedical Imaging, Massachusetts General Hospital, Harvard Medical School, Cambridge, USA
8 Computer Science and Artificial Intelligence Lab, Massachusetts Institute of Technology, Cambridge, USA
9 Department of Radiology, Ludwig-Maximilians-University, Munich, Germany
10 Department of Medicine I, Medical University of Vienna, Vienna, Austria

*Address correspondence to:
Karl-Heinz Nenning, karl-heinz.nenning@meduniwien.ac.at
Georg Langs, georg.langs@meduniwien.ac.at

Patient cohort

ID	Gender	Age	Location	Hemisphere	Tumour Volume (enhancing + necrosis)	Follow-up acquisitions
Pat01	male	77	Temporal	Left	15.55 cm^3	8
Pat02	female	64	Parietal	Right	58.40 cm^3	6
Pat03	male	51	Frontal	Right	55.22 cm^3	6
Pat04	male	58	Frontal	Left	20.84 cm^3	4
Pat05	female	50	Frontal	Left	38.43 cm^3	2
Pat06	male	61	Temporal	Right	50.55 cm^3	1
Pat07	female	78	Frontal	Right	9.51 cm3	-
Pat08	male	73	Limbic	Right	44.05 cm^3	-
Pat09	male	65	Occipital	Right	67.33 cm^3	-
Pat10	female	71	Frontal	Right	22.22 cm^3	-
Pat11	male	48	Parietal	Left	32.18 cm^3	-
Pat12	female	67	Frontal	Left	11.87 cm^3	-
Pat13	male	55	Temporal	Left	32.29 cm^3	-
Pat14	male	53	Frontal	Right	29.12 cm^3	-
Pat15	female	47	Parietal	Left	17.54 cm^3	-

Supplementary Table 1. Overview of the study cohort, primary tumour location, tumour extent and the number of longitudinal follow-up scans.

Functional network anomaly follows functional rather than spatial distance to the tumour

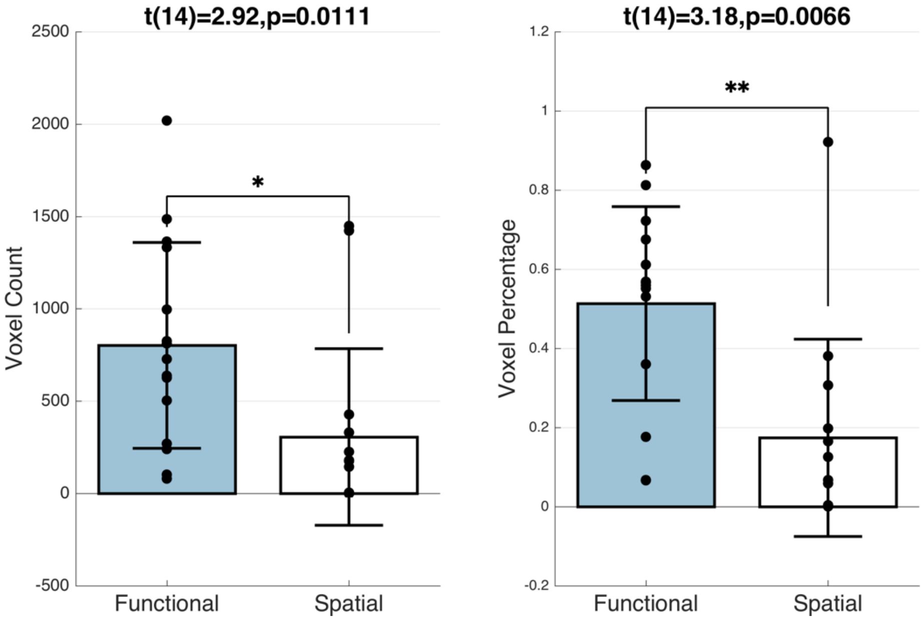

Supplementary Figure 1. A paired t-test between the number of voxels (left), as well as their percentage of the tumour volume (right), revealed a significantly higher number of tumour voxels where anomaly relates to functional proximity rather than spatial distance. Figure created with MATLAB 2014a (www.mathworks.com) and Microsoft Office PowerPoint 2016 (www.microsoft.com).

Overlap between lesion maps and anomaly scores

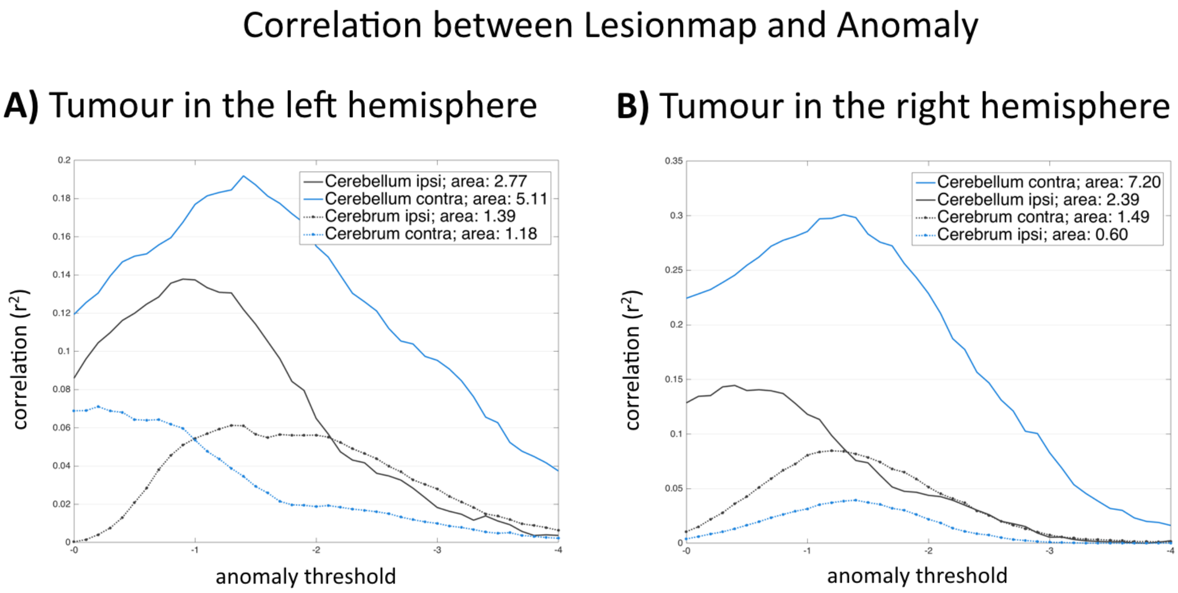

Supplementary Figure 2. Correlation between lesion-map and anomaly concordance. **A)** Patients with a tumour located in the left hemisphere showed a higher correlation between lesion-map and anomalies in the contralateral (right) cerebellum (paired t-test: $t=15.92$, $p < 0.0001$). Correlation analysis with a varying threshold resulted in an area of 5.11 for the contralateral compared to 2.77 for the ipsilateral cerebellum. **B)** The same effect was observed in patients with a tumour located in the right hemisphere (paired t-test: $t=11.77$, $p < 0.0001$), where the contralateral (left) cerebellum showed an area of 7.20 and the ipsilateral cerebellum an area of 2.39. Figure created with MATLAB 2014a (www.mathworks.com) and Microsoft Office PowerPoint 2016 (www.microsoft.com).

Decrease of network anomaly at first follow-up after glioblastoma surgery

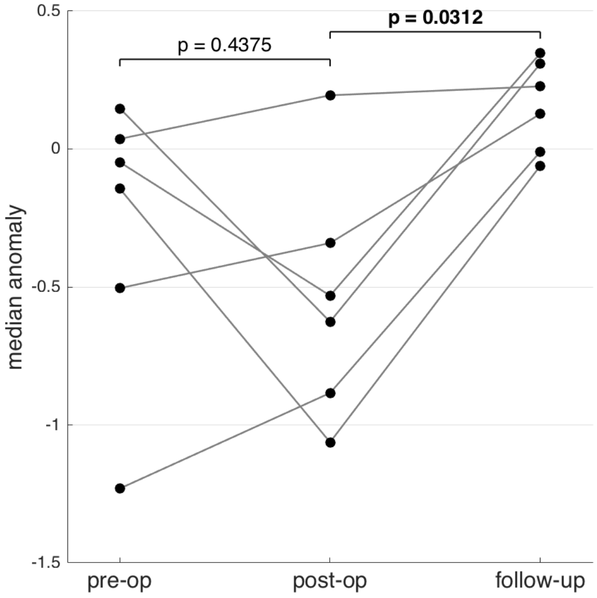

Supplementary Figure 3. Results indicate no significant difference in the patients' median whole-brain anomaly before and after surgery, while there is a reduced anomaly at first follow-up. Figure created with MATLAB 2014a (www.mathworks.com) and Microsoft Office PowerPoint 2016 (www.microsoft.com).

Anomaly was not influenced by connectivity to tumour voxels and hemisphere

A) Pairwise correlation of anomaly maps with and without tumour voxels for calculation.

B) Lateralization of anomaly scores

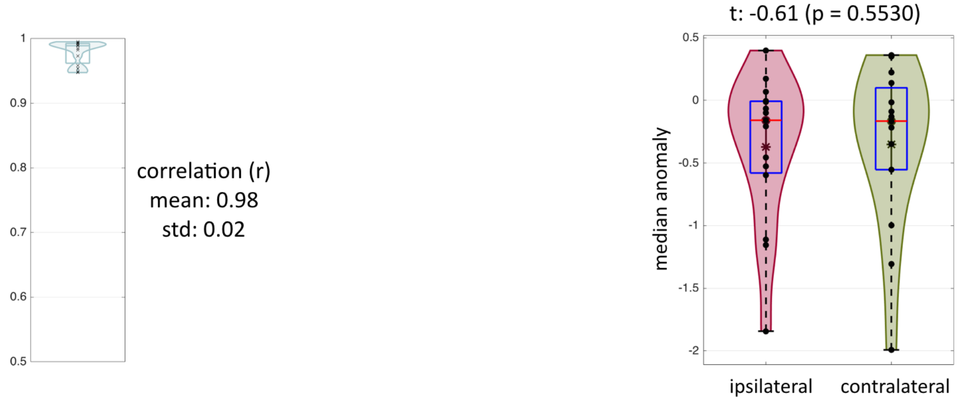

Supplementary Figure 4. A) Patient-specific anomaly maps excluding all tumour voxels were highly similar to anomaly maps including tumour voxels, with an average similarity of r = 0.98 (± 0.02; p < 0.0001). **B)** Analysis of the lateralization of the anomaly scores did not show a significant difference between the ipsilateral and contralateral tumour hemisphere (t = -0.61, p = 0.5530). Figure created with MATLAB 2014a (www.mathworks.com) and Microsoft Office PowerPoint 2016 (www.microsoft.com).

Anomaly precedes tumour recurrence

In Patient 01, we observed tumour recurrence and continued progression at the third follow-up scan at 7 months after surgery. We observed that the anomaly score, measured at the second follow-up scan 5 months after surgery coincided with the future emerging tumour at the third follow-up. Areas that progressed to enhancing tumour in the third follow-up after surgery showed a significantly higher anomaly in the prior examination ($t = 88.39$; $p < 0.0001$; Hedges' g effect size = 1.42) compared to all other voxels in a 1 cm vicinity surrounding the tumour.

In Patient 02, tumour recurrence occurred at the second follow-up examination, 4 months after surgery, with corresponding increased anomaly in the previous acquisition ($t = 15.21$; $p < 0.0001$; Hedges' g effect size = 0.36) in regions of future tumour growth compared with the affected tumour vicinity, which showed no emerging tumour.

Patient 03 exhibited tumour progression at the sixth follow-up scan at 13 months after surgery. Although overall less functional anomaly was observed at the fifth follow-up it was still significantly higher ($t = 29.39$; $p < 0.0001$; Hedges' g effect size = 0.48) compared to the non-recurrent tumour vicinity.

In Patient 04, the tumour recurred at the first follow-up scan, and 4 months after initial surgery. Anomaly score at the recurrence region showed significant elevation at the post-operative fMRI acquisition ($t = 39.13$; $p < 0.0001$; Hedges' g effect size = 0.5).

Patient 05 showed rapid emerging recurrence contralateral to the primary tumour one month after surgery. Visually, the growing tum tumour or showed functional anomaly, but no significantly higher anomaly scores were found between emerging tumour voxels and the non-recurrent tumour vicinity ($t = -1.57$; $p = 0.1128$; Hedges' g effect size = 0.02).

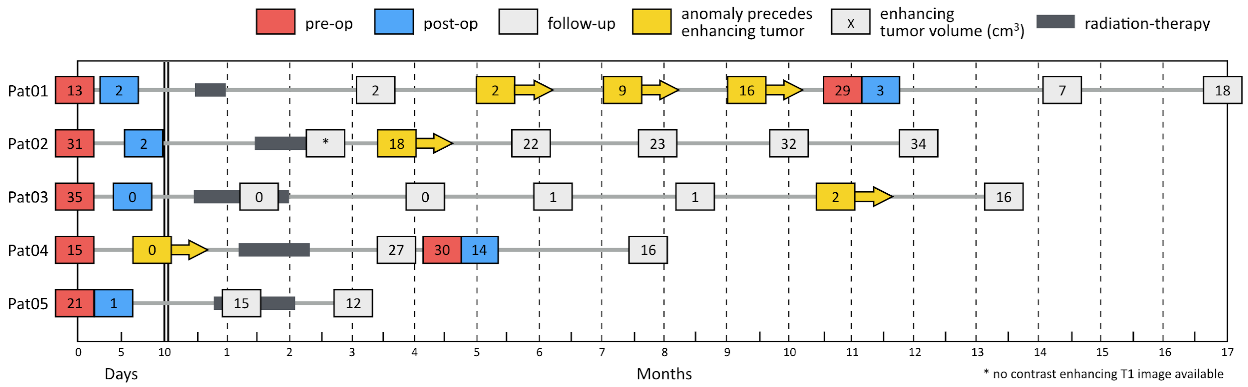

Supplementary Figure 5. Overview of the patient-specific longitudinal MRI acquisitions. The intervals of longitudinal MRI acquisitions, with the time-point specific enhancing tumour volume, are depicted as squares and pre- and post-surgical scans are marked as red and blue. Time-points were anomalies precede tumour recurrence are highlighted as yellow. Figure created with Microsoft Office PowerPoint 2016 (www.microsoft.com).
